# Genomic instability and *CCNE1* amplification as emerging biomarkers for stratifying high-grade serous ovarian cancer

**DOI:** 10.3389/fonc.2025.1633410

**Published:** 2025-08-06

**Authors:** Elena Conca, Daniele Lorenzini, Emanuela Minna, Luca Agnelli, Matteo Duca, Marco Gentili, Beatrice Bodini, Maggie Polignano, Mara Mantiero, Silvia Damian, Andrea Devecchi, Gianpaolo Dagrada, Rita Carminati, Alice Ardore, Francesca Barbetta, Nathalia Brito Da Chuna, Andrea Guerrizio, Adele Busico, Iolanda Capone, Alberta Piccolo, Elena Tamborini, Federica Perrone, Massimo Milione, Biagio Paolini, Andrea Vingiani, Francesco Raspagliesi, Filippo De Braud, Giancarlo Pruneri

**Affiliations:** ^1^ Department of Diagnostic Innovation, Pathology Unit 2, Fondazione IRCCS Istituto Nazionale Dei Tumori, Milano, Italy; ^2^ Department of Oncology and Hemato-Oncology, University of Milan, Milano, Italy; ^3^ Department of Medical Oncology, Fondazione IRCCS Istituto Nazionale Dei Tumori, Milano, Italy; ^4^ Department of Pathology and Laboratory Medicine, Pathology Unit 1, Fondazione IRCCS Istituto Nazionale Dei Tumori, Milano, Italy; ^5^ Gynecologic Oncology Unit, Fondazione IRCCS Istituto Nazionale dei Tumori, Milano, Italy

**Keywords:** high-grade serous ovarian cancer (HGSC), comprehensive genomic profiling (CGP), homologous recombination deficiency (HRD) score, CCNE1 amplification, precision oncology cancer

## Abstract

**Introduction:**

Ovarian cancer (OC) is one of the leading causes of cancer-related death in women worldwide. Treatment with PARP-inhibitors has significantly improved survival in patients with high-grade serous cancer (HGSC) bearing *BRCA1/2* mutations (~22% of the cases), and/or homologous recombination deficiency (HRD, ~50%). Unfortunately, limited therapeutic alternatives are available for *BRCA1/2* wild type/HR proficient HGSC patients, who usually exhibit resistance to standard treatments and poor prognosis.

**Methods:**

Herein, we present the results of a comprehensive genomic profiling (CGP) analysis using the Oncomine Comprehensive Assay® (OCA) Plus in a consecutive retrospective cohort of 102 HGSC patients characterized in our institution.

**Results:**

Genomic instability, measured by Genomic Instability Metric (GIM) >16, was found in 40% of the cases and was significantly associated with *BRCA1/2* mutations (p=0.009), with a better prognosis in terms of recurrence-free survival (p=0.01). *CCNE1* amplification was observed in 29% of cases and was negatively correlated with BRCA1/2 mutations (p=0.001), without any association with GIM, supporting *CCNE1* as a strong and independent driver of tumorigenesis. Additionally, *CCNE1* amplification was validated with fluorescent in situ hybridization (FISH), supporting the analytical robustness of NGS data (rho=0.93), and investigated by immunohistochemistry (IHC), revealing that *CCNE1* protein overexpression was observed in the absence of gene amplification in 45% of cases.

**Discussion:**

Our real-world study supports the clinical utility of the GIM metric and the analytical validity of *CCNE1* amplification, a new promising biomarker for personalizing treatment in HR proficient HGSC patients. The discordance between *CCNE1* amplification and protein expression raises intriguing questions about the mechanisms of *CCNE1*-driven tumorigenesis and warrants further investigation.

## Introduction

Ovarian cancer (OC) accounts for 3% of all cancers in women and represents the deadliest gynecologic malignancy, with 294,000 new diagnoses and 198,000 deaths per year worldwide. Despite advancements in disease management, the 5-year survival rate for OC patients remains below 50% in most countries (1). This is largely attributed to the advanced stage at which the disease is typically diagnosed, the lack of solid screening tests (2), and the frequent occurrence of chemoresistance (3). Primary debulking surgery followed by platinum-based chemotherapy (CT) is the standard therapeutic approach for advanced stage disease (III and IV), while maintenance treatment with bevacizumab and/or poly (ADP-ribose) polymerase inhibitors (PARPi) is based on molecular and clinical characteristics (National Comprehensive Cancer Network (NCCN) Clinical Practice Guidelines in Oncology, Version 3.2024).

OC is a highly heterogeneous disease with five major etiologically and genetically distinct histological types of ovarian carcinomas bearing specific molecular features, clinical behavior, response to treatment, and outcome (4). High-grade serous carcinoma (HGSC) is the prevalent histology (70%–75%), accounting for almost 80% of the deaths (5). HGSC harbors inactivating mutations in the tumor suppressor gene *TP53* in >95% of the cases and germline or somatic *BRCA1/BRCA2* gene variants, which are frequently associated with homologous recombination deficiency (HRD) in up to 22% of the cases (6). *BRCA1/2* mutations represent both a prognostic factor and a predictive marker of response to platinum-based chemotherapy and to PARPi (7). Moreover, patients carrying a germline mutation in *BRCA1/2* have increased cancer risk, which may also involve their relatives (8). For these reasons, the American Society of Clinical Oncology guidelines stated that germline or somatic *BRCA1/2* testing should be performed in all patients with epithelial OC (8, 9). Along this line, the European Society for Medical Oncology guidelines stated that all patients with high-grade OC should be tested for germline and/or somatic *BRCA1/2* mutations at diagnosis, and HRD testing is recommended in advanced cases (10). Several assays have been developed to evaluate *BRCA1/2* and HR status (11). The Food and Drug Administration (FDA)-approved Myriad MyChoice CDx panel predicts HRD using a genomic instability score (GIS) computed by integrating loss of heterozygosity (LOH), large-scale state transitions (LSTs), and telomeric–allelic imbalance (TAI) (7, 11). *BRCA1/2* mutation and HRD are currently the only biomarkers approved for targeted therapeutic approaches with PARPi in HGSC patients; indeed, the FDA recently approved the Myriad MyChoice assay for HRD detection (12). HRD is a multifaceted biomarker, arising from a combination of diverse genomic alterations, that requires a comprehensive and complex analysis for its accurate definition (12). Alternative tests assessing HRD through different algorithms have been recently implemented, including FoundationOne^®^ CDx (Foundation Medicine), TruSight Oncology 500 HRD (Illumina), SOPHiA DDM HRD (SOPHiA Genetics), and AmoyDx HRD Focus Panel (AmoyDx) (13, 14). Particularly, Oncomine Comprehensive Assay Plus (Thermo Fisher Scientific) was recently implemented with HRD analysis by means of Genomic Instability Metric (GIM) computing, whose algorithm has already undergone extensive validation (14–17). In addition to HRD analysis, approximately 500 other genes are characterized for their mutational and copy number status, making OCA Plus one of the more complete target next-generation sequencing (NGS) assays available.

The mutational spectrum of HGSC also includes *BRAF*, *NF1*, *ATR*, *ATRX*, *PIK3CA*, *RB1*, *CDK12*, *FAT3*, *CSMD3*, and *GABRA6* mutations, albeit at lower rates (1%–6%) (18, 19), with many of these alterations currently recognized as therapeutics targets in other solid tumors or under investigation in experimental clinical trials (20). In addition, HGSC is a chromosomally unstable malignancy commonly bearing amplifications in the *CCNE1* (~20%), *MYC* (~30%), and *MECOM* (~25%) genes (21). *CCNE1* amplification particularly represents one of the most promising targets for *BRCA1/2* wild-type, HR-proficient HGSC patients (6). In this regard, AZD1775 (Adavosertib), a WEE1 kinase inhibitor operating downstream of *CCNE1*, or the CDK2 inhibitors INCB123667 and BLUE-222, which directly prevent the CCNE1-CDK2 complex formation, are currently under clinical investigation for anticancer activity in HGSC patients carrying *CCNE1* amplification or protein overexpression (22–24). Recently, other cell-cycle inhibitors, lunresertib and azenosertib, respectively targeting PKMYT1 and WEE1, have been introduced in clinical trials concerning ovarian cancer and many other solid tumors (25–27).

Collectively, these data support the rationale for including *CCNE1* among the biomarkers to be tested routinely in advanced/metastatic cancer patients.

No real-world series from clinical practice using a single Comprehensive Genomic Profiling (CGP) commercially available target DNA panel has been reported to date. For example, in 2019, Zhong et al. (28) investigated a cohort of 88 individuals using a custom panel, but they did not describe any *CCNE1* amplification. Dumur et al. (16) validated the OCA Plus panel in a pan-cancer study including generically defined ovarian cancer.

In this scenario, we report a complete molecular characterization of 102 consecutive HGSC, carried out for both optimizing patients’ stratification with currently available therapies and uncovering targets for enrollment in clinical trials in the context of our institutional molecular tumor board (29).

## Patients and methods

### Patients and sample collection

The study comprises a cohort of 102 consecutive patients with HGSC who were tested using CGP at the Department of Diagnostics Innovation of Fondazione IRCCS Istituto Nazionale dei Tumori (Milan, Italy) from March 2022 to October 2023. The study was performed in compliance with laws and institutional guidelines and has been approved by the internal Independent Ethics Committee (protocol INT 191/24, approved on 09/17/2024). All patients signed an informed consent prior to NGS data analysis. The NGS test was usually performed at cancer diagnosis, except for patients referred to our institution at disease relapse. Outcome data were retrieved from electronic health records. For outcome analysis, the following data were collected: diagnosis date, platinum-based CT starting date, maintenance therapy, and relapse date. Tumor samples were obtained from formalin-fixed paraffin-embedded (FFPE) samples. Hematoxylin–eosin sections were revised by expert pathologists to define the tumor area and cancer cell percentage. The tumor area was manually macro-dissected when necessary. DNA was extracted as previously reported (30), DNA quality was assessed by means of the 4200 TapeStation System (Agilent, CA, United States), and DNA quantity was established using Qubit High Sensitivity dsDNA quantification assay kit (Thermo Fisher Scientific, MA, United States, catalog Q32851).

### Library preparation, sequencing, and analysis

CGP was performed using the commercial Oncomine Comprehensive Assay (OCA) Plus (Thermo Fisher Scientific, MA), which allows the detection of gene mutations, copy number variation (CNV), microsatellite instability (MSI), and tumor mutational burden (TMB) in 500 cancer-related genes. Libraries were generated according to the manufacturer’s instructions, quantified by the Ion Library TaqMan Quantification kit (Thermo Fisher Scientific, catalog #4468802), and diluted to a final concentration of 60 pM. Sequencing was performed on the Ion GeneStudio S5 System (Thermo Fisher Scientific) loading Ion 550 chips to obtain at least 20 million reads. Sequencing output was evaluated on the Torrent Suite 5.18.1 (Thermo Fisher Scientific) using the Coverage Analysis plugin. Library uniformity and on-target metric thresholds for quality acceptance were set to 90% and 2,000× medium coverage per case, respectively. BAM files were generated using the Variant Caller plugin of the Torrent Suite and analyzed by Ion Reporter Software (IRS) 5.20.2.0 (Thermo Fisher) with the Oncomine Comprehensive Plus 5.20 w3.1 DNA workflow (default options). The variant call format (VCFs) obtained were loaded on openCRAVAT (31), and the output variants were filtered by i) Sequence Ontology, ii) clinical significance, and iii) 1000 Genomes population frequency < 0.0001. The resulting variants were manually checked using the Integrative Genomics Viewer, Broad Institute (32), to filter out potential false-positive calls. The clinical relevance of gene variants was annotated by referring to available public databases ClinVar (https://www.ncbi.nlm.nih.gov/clinvar/), cBioPortal (https://www.cbioportal.org/), and BRCA Exchange (https://brcaexchange.org/) and finally classified according to the American College of Medical Genetics and Genomics and the Association for Molecular Pathology (ACMG–AMP) guidelines. Pathogenic and likely pathogenic variants were considered for potential therapeutic options by standard care or targeted therapies, following the European Society of Medical Oncology (ESMO) indications (33). CNV analysis was performed on IRS (Ion Reporter Software 5.20 User Guide, MAN0028321). Copy number estimate reliability was evaluated using the Median of the Absolute values of all Pairwise Differences (MAPD > 0.5 as mandatory threshold for CNV analysis), calculated for each sample, as well as the CNV-pValue, calculated for each call. CNV calls with p-value > 0.00001 were considered not reliable (34) and then excluded.

### Fluorescence *in situ* hybridization

Fluorescence *in situ* hybridization (FISH) was carried out in cases bearing *CCNE1* copy number gains by NGS. The Abnova CCNE1/CEN19p dual-color FISH Probe kit, containing the Spectrum Red-labeled *CCNE1* probe and the Spectrum Green-labeled Centromere 19 probe (#16032995), was used in FFPE sections. FISH slides were evaluated using a Leica DM 6000B (Wetzlar, Germany) microscope at ×100 magnification. A minimum of 50 tumor nuclei from the formalin-fixed tissue were scored. The images were captured using the CytoVision software (v. 7.0, Leica). Samples with a ratio of *CCNE1* to chromosome 19 centromere signals > 2, as well as samples with multiple copies of the *CCNE1* gene arranged in clusters, were considered amplified as described by Varella-Garcia et al. (35).

### Immunohistochemistry

Immunohistochemistry (IHC) was carried out to investigate the protein expression beyond gene copy gain. Three-micrometer FFPE sections were placed on polarized glass slides; antigen retrieval was performed at high pH and 96°C for 15 minutes. The anti-CCNE1 antibody (clone HE12, sc-247, Santa Cruz, CA, USA) and the Dako Autostainer Link 48 were used. Testis and kidney slides were used as positive (germ cells and glomeruli, respectively) and negative (Leydig cells and tubules, respectively) controls. Staining was blindly evaluated by two pathologists and assessed using the H-score; neoplastic cells were divided into four groups (negative, weak, moderate, and strong) based on the expression level of CCNE1 in both the nucleus and cytoplasm. The H-score was calculated as a weighted average of the percentages of weak staining (weight = 1), moderate staining (weight = 2), and strong staining (weight = 3), with a range of 0–300. Discordant cases were further discussed until an agreement was reached. Based on the H-score distribution, cases were further assessed as positive or negative. The H-score threshold was set at 120 by calculating the lowest point of the bimodal distribution of H-score (120.9897) (**Supplementary Figure S1**).

### Statistical analysis

All the associations of categorical variables (*CCNE1* amplification, *BRCA1/2* mutation, and GIM positivity) with numeric variables (GIM scores, %LOH, total number of SNV, TMB, total number of mutations, and CNVs in the HR gene) were evaluated using the Mann–Whitney test. Categorical variables (genomic instability status and BRCA1/2 mutation presence, *CCNE1* amplification and IHC status, *CCNE1*, *BRCA1/2* status, HRD, and disease relapse) were compared by Fisher’s test. Correlation analysis was performed using Spearman’s test. Recurrence-free survival (RFS) was analyzed using the log-rank test and the Kaplan–Meier method. Odds ratio (OR) values and their 95.5% confidence intervals were extracted from a multivariate logistic regression model fitted to individual-level data. All the analyses and visualizations were performed using R Version 4.3.1 (R Foundation for Statistical Computing).

## Results

Clinico-pathological characteristics are detailed in **Table 1**. The median patient age was 63 years (range 36–88). According to the International Federation of Gynecology and Obstetrics (FIGO) (27) staging, 63.89% and 23.61% of the patients had stage III and stage IV diseases, respectively. Correlations between molecular data and outcome were available for 61 out of the 102 patients included in the study. Most patients (76/102, 74.51%) were diagnosed and treated in our institute, whereas the remaining (26/102, 25.49%) were outpatients who were referred for genomic profiling.

**Table 1:** Clinico-pathological characteristics.

Number of patients	102
Median age	63
Material provenience	
Internal	74
External	28
FIGO stage	
I	7
II	1
III	45
IV	18
Unknown	31
Recurrence-free survival	
Recurred	41
Not recurred	27
Unknown	34
Treatment	
Maintenance	47
Not maintenance	21
Unknown	34
Site	
Ovary/uterus	35
Others	67

FIGO, International Federation of Gynecology and Obstetrics.

### CGP analysis of HGSC confirmed high prevalence of pathogenic mutations

**Figure 1:**
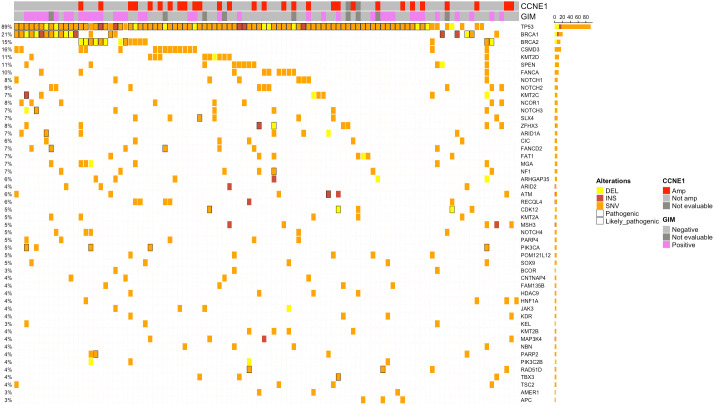
Oncoplots showing the top 50 altered genes by mutations. Only patients harboring alterations in selected genes are reported. Samples are represented by columns, and genes are represented by rows. Pathogenic/likely pathogenic alterations are marked in black. Indications about the Genomic Instability Metric (GIM) *CCNE1* amplification status were also added. Amp, amplified; Not amp, not amplified; DEL, deletion; INS, insertion; SNV, single-nucleotide variation.

**Figure 1** shows an overview of the mutational spectrum of the HGSC patients included in this study. Overall, 968 variants were detected in 102 cases (median, 8; range, 3–50), with a median TMB of 3.83 (range, 0–51.08). As expected, the commonest mutated genes were *TP53* (94/102, 92.15%, with 81.37% pathogenic/likely pathogenic variants), *BRCA1* (22/102, 21.57%, with 18/102, 17.64%, pathogenic/likely pathogenic), and *BRCA2* (16/102, 15.68%, with 9/102, 8.82% pathogenic/likely pathogenic) (**Figure 1**). Overall, pathogenic/likely pathogenic somatic *BRCA1/2* mutations were detected in 27 cases (26.47%), with 10 of them (37.07%) confirmed as germline.

Remarkably, in 2/102 cases (1.96%), a p.Y220C variant of the *TP53* gene was found. Notably, the inactivating mutation p.Y220C in p53 protein is also an emerging agnostic target, representing a potential therapeutic chance in an ongoing basket trial (19). In addition to *BRCA1/2*, the homologous recombination repair (HRR) genes were mutated in 23 cases (22.55%), mostly *ATM* (5.88%), *FANCA* (9.80%), *NBN* (3.92%), *FANCD2* (6.86%), *CDK12* (4.90%), and *BRIP1* (2.94%). Notably, mutations were found in other potentially targetable genes, including *PIK3CA* (4/102, 3.92%), *CDK12*, *PTEN*, *ARID1A*, or *BRAF* (1/102 each, 0.98%).

The GIM was generated by calculating the unbalanced copy number changes in 46 genes associated with HR response, determined using genomic segmentation and yielding values ranging from 0 to 100. According to the manufacturer’s guidelines, a GIM score ≥ 16 indicates HRD as a pathogenic—and therefore druggable—condition. Sample-level LOH percentage (%LOH) is the percentage of genomic segments with LOH detected. It is calculated as the sum of the sizes of the genomic segments with LOH detected/total size of genomic segments assessed for LOH.

In 93 cases (91.17%), MAPD scores were >0.5 and were evaluable for CNV analysis as well as for GIM detection. Of these, 37 (39.78%) cases had a positive GIM score (≥16). There was a significant association between GIM score and *BRCA*1/2 mutation (p = 0.009142) or increased LOH percentage (p = 2.721e−05, **Supplementary Figure S1**). Eight *BRCA1/2*-mutated patients had a negative GIM score.

### CCNE1 amplification is mutually exclusive with *BRCA1/2* alteration and independent of HRD-related molecular signature

**Figure 2:**
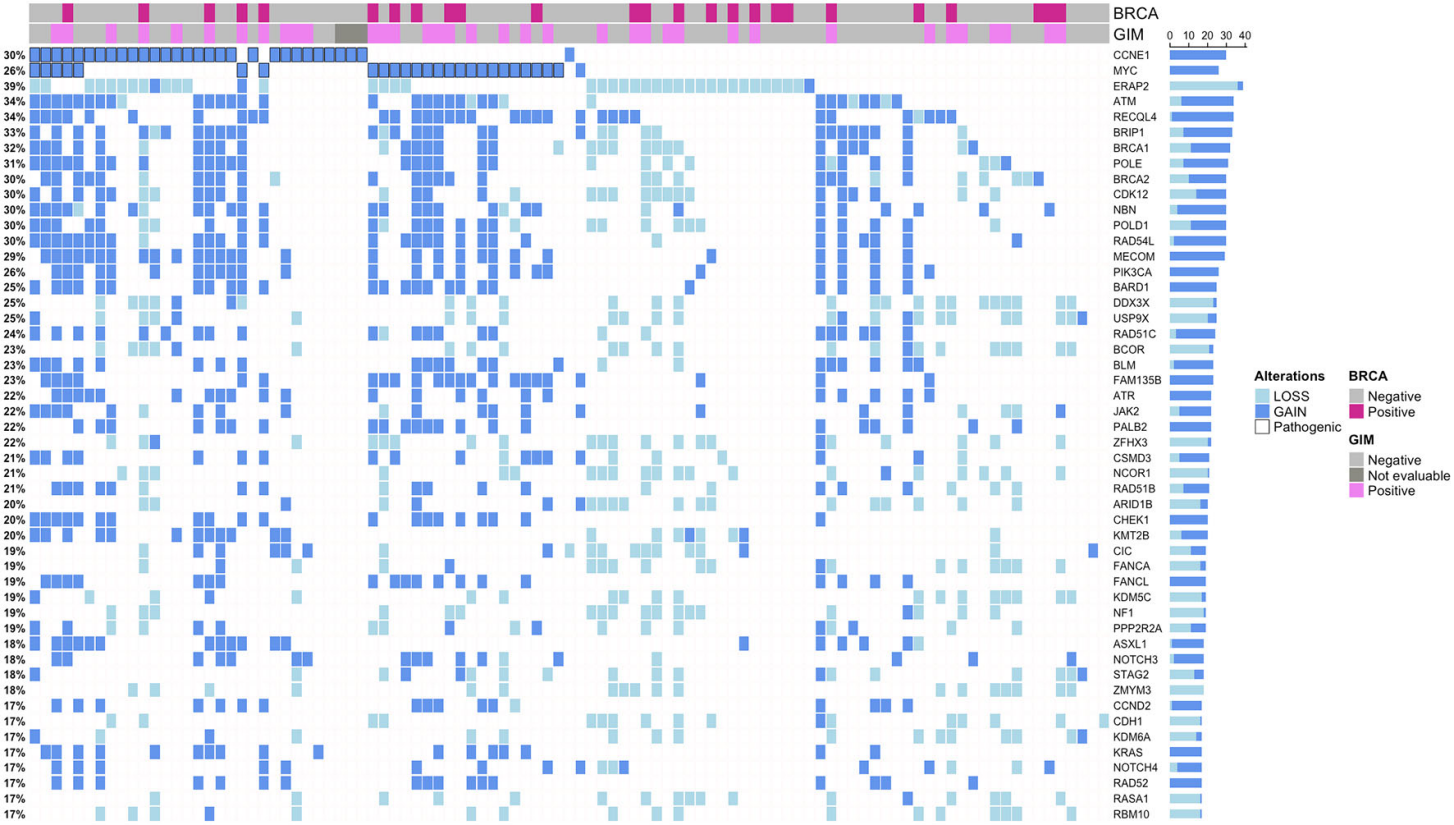
Oncoplots showing the top 50 altered genes by copy number variations. Only patients harboring alterations in selected genes are reported. Samples are represented by columns, and genes are represented by rows. Pathogenic/likely pathogenic alterations are marked in black. Indications about Genomic Instability Metric (GIM) and *BRCA1/2* status were also added. Amp, amplified; Not amp, not amplified; DEL, deletion; INS, insertion; SNV, single-nucleotide variation.

**Figure 3:**
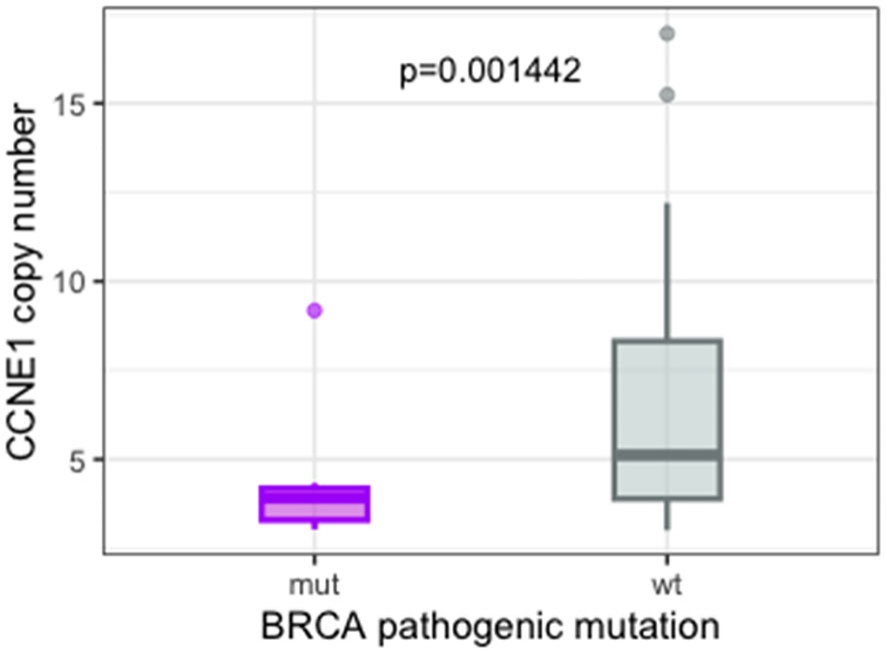
Boxplot showing the inverse correlation between pathogenic *BRCA1/2* mutation and *CCNE1* amplification detected by NGS. NGS, next-generation sequencing.

CNVs (**Figure 2**) occurred most frequently in the HRR genes, which represented nine of the 15 top altered genes. Copy number losses were observed in the *ERAP2* (39.78%), *BRCA1* (13.97%), *BRCA2* (10.75%), *BRIP1* (9.68%), *ATM* (5.37%), *POLE* (7.52%), *CDK12* (17.20%), and *POLD1* (4.3%) genes. The most common genes carrying copy number gains were *CCNE1* (29.03%), *MYC* (26.88%), *MECOM* (31.18%), *BARD1* (29.03%), *PIK3CA* (26.88%), *KRAS* (17.02%), *JAK2* (21.50%), *FAM135B* (23.65%), *ATR* (30.10%), *NOTCH3* (15.05%), *CCND2* (15.05%), and *ASXL1* (18.28%). Our CNV analysis was focused on genes potentially actionable with personalized treatments. *CCNE1* amplification was found in 27/93 cases (29.03%, **Figure 2**), and it was negatively associated with *BRCA1/2* mutation (p = 0.001442, **Figure 3**). Specifically, only two cases showed both *BRCA1/2* mutations and *CCNE1* amplification. The association between *CCNE1* amplification and other molecular features related to genome instability, including GIM score, TMB, and LOH, was also tested: just a weak correlation between *CCNE1* amplification and TMB was observed, with a median TMB of 3.10 and 4.75 in the amplified and non-amplified cases (p = 0.01813, **Supplementary Figure S2**).

Altered actionable genes behind *BRCA1/2* or *CCNE1* were rare, appeared to be more often altered in the HRD setting, and most rarely co-occurred with *CCNE1* amplification (2/38 HRD-positive *PIK3CA*-mutated, 1/38 HRD-positive *BRAF*-mutated, 1/38 HRD-positive *CDK12*-mutated, 1/27 *CCNE1*-positive *PIK3CA*-mutated, and 1/27 *CCNE1*-positive *CDK12*-mutated). Finally, pathogenic or likely pathogenic mutations in potentially actionable genes were also rare among *CCNE1*-negative/HR-proficient cases, being identified in eight out of 50 cases (16%). Specifically, these included two *CDK12* mutations, one *BRAF* mutation, one *KRAS* mutation, two *PIK3CA* mutations, one *NF1* mutation, and one *PTEN* mutation.

### *CCNE1* amplification by NGS is robustly validated by fluorescence *in situ* hybridization

**Figure 4:**
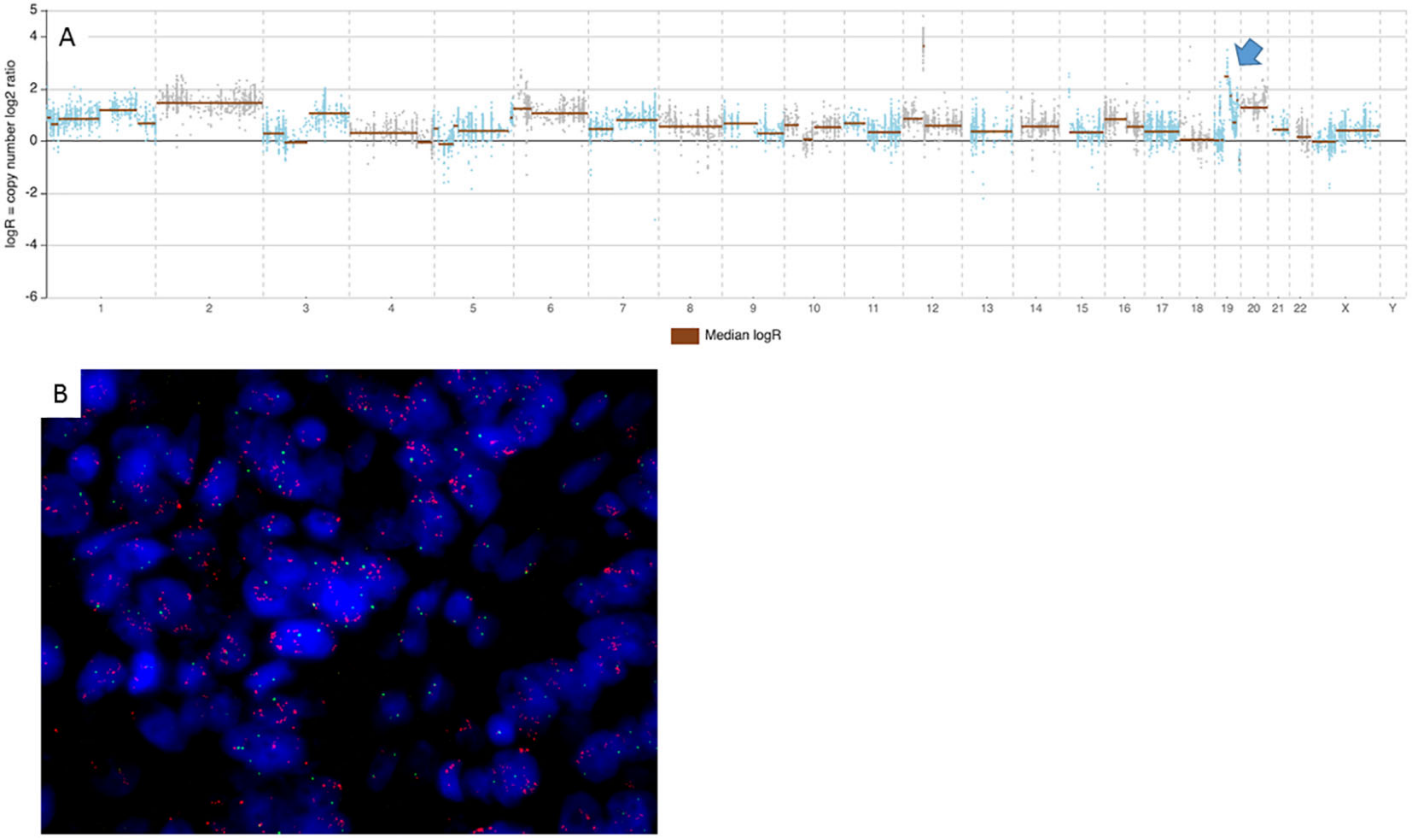
NGS and FISH *CCNE1* pattern in an amplified case. **(A)** Top panels showing log2 ratios of the copy number estimates by NGS assay for each amplicon (blue and brown points). Brown horizontal bars represent genomic segmentation resulting from amplicons with similar log2 ratios clustered together. The blue arrow indicates the segment localized on CytoBand 19q12, corresponding to *CCNE1* gene amplicons. The image shows a case with focal amplification of *CCNE1*, confirmed by FISH in the picture below **(B)**, where green and red signals refer to centromere and *CCNE1* gene, respectively. NGS, next-generation sequencing; FISH, fluorescence *in situ* hybridization.

Given the clinical interest regarding the *CCNE1* gene as an emerging therapeutic target, *CCNE1* NGS data were validated using FISH (**Figure 4**). FISH was carried out in 42 cases, including 21/27 NGS amplified cases, 15/64 NGS non-amplified cases, and 6/9 cases for which CNV NGS analysis had failed. In all but one of the 35 cases analyzable and evaluable with both techniques, FISH confirmed the NGS results.

In most of the amplified cases, FISH *CCNE1* signals were arranged in clusters, which makes it impossible to precisely enumerate the *CCNE1* copy number by calculating a ratio *CCNE1*/cen19. In those cases, an estimate based on the size of the clusters was offered: smaller clusters were estimated to contain five to eight gene copies, and large clusters contained >10 or >20 copies according to their size (**Supplementary Table S1**).

FISH copy number values, thus estimated, together with NGS ones, are reported in **Supplementary Table S2**. A robust linear association was observed between NGS and FISH copy number values as assessed by Spearman’s correlation test (rho = 0.93, p = 7.261e−15) (**Table 2**).

**Table 2:** NGS/FISH result contingency table.

	Amp FISH	Not amp FISH	Na/ne FISH
Amp NGS	19	0	8
Not amp NGS	1	15	48
Na/ne NGS	2	3	6

NGS, next-generation sequencing; FISH, fluorescence *in situ* hybridization; Amp, amplified; Not amp, not amplified. Na/ne: not available/not evaluable.

### Immunohistochemistry for CCNE1 unravels CCNE1 expression in the absence of amplification and irrespective of NGS copy number

**Figure 5:**
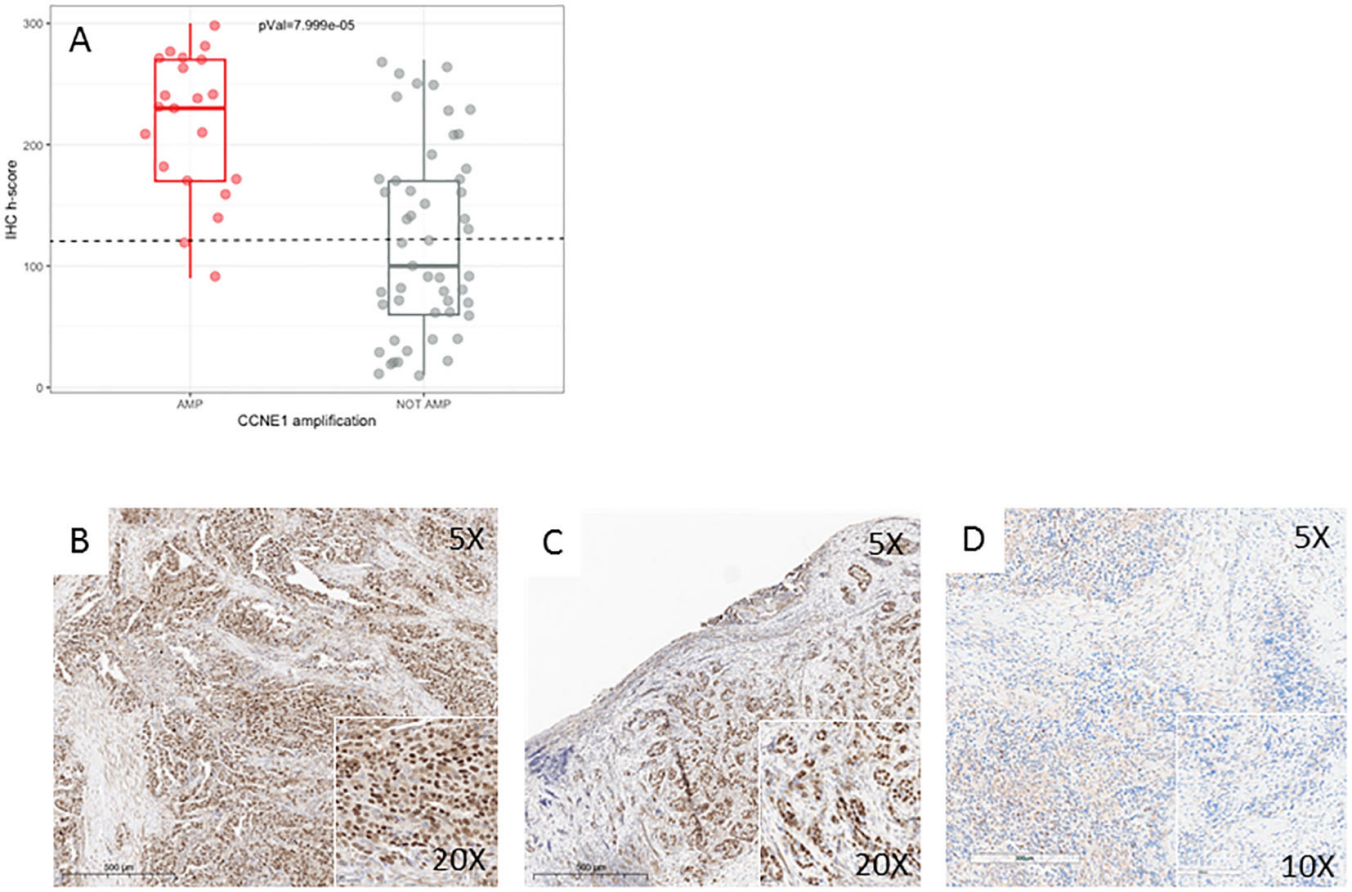
CCNE1 immunohistochemistry. **(A)** Boxplot showing the positive correlation between *CCNE1* amplification results, detected by both NGS and FISH, and protein expression, detected by IHC. **(B–D)** Representative IHC staining of CCNE1 in three HGSC cases. **(B)** HGSC with *CCNE1* amplification and positive IHC. **(C)** HGSC with no *CCNE1* amplification and positive IHC. **(D)** HGSC with no *CCNE1* amplification and a negative IHC. Original magnification: ×5 main image and ×20–×10 boxes. HGSC, high-grade serous ovarian carcinoma; IHC, immunohistochemistry; NGS, next-generation sequencing; FISH, fluorescence *in situ* hybridization.

Forty-three out of the 73 (58.9%) cases analyzed by IHC (**Table 3**) were classified as positive based on the threshold described above. A statistically significant correlation was found between *CCNE1* amplification and positivity by IHC (p = 7.999e−5, **Figure 5A**). In particular, most *CCNE1* amplified cases were positive by IHC (19/21, 90.5%). In contrast, high levels of CCNE1 immunoreactivity were found in 23/51 of non-amplified cases (45%, **Figure 5B**). These results were confirmed by Spearman’s rank correlation analysis conducted between NGS copy number and H-score values, showing a moderate but statistically significant positive association (rho = 0.50, p = 7.887e−05). Then, the behavior of CCNE1 expression assessed by IHC in patients showing low-level *CCNE1* gains (three to five copies) was tested, and 8/12 cases (67%) were found, actually resembling the percentage observed in patients showing normal *CCNE1* copy number (17/27, 63%). No significant differences in terms of *BRCA1/2* mutations, GIM score, or other markers present were observed between CCNE1-expressing and non-expressing groups.

**Table 3:** Patients’ molecular characteristics.

		CCNE1 copy number status		*BRCA1/2* status		Genomic instability status	
		Amplified (>5 copies)	Not amplified (<5 copies)	BRCAwt	BRCAm*	Negative (GIM < 16)	Positive (GIM > 16)
Total mutations		Median = 8	Median = 9	Median = 8	Median = 11	Median = 8.5	Median = 8.5
TMB		Median = 3.10	Median = 4.75	Median = 3.79	Median = 5.67	Median = 3.79	Median = 4.74
LOH%		Median = 17.23	Median = 21.29	Median = 14.58	Median = 24.63	Median = 13.33	Median = 34.00
MSI status	High MSI	0	2	0	2	1	1
	MSS	27	61	72	25	54	38
	Not evaluable	0	2	3	0	0	4
FISH	Negative	0	15	14	4	10	8
	Positive	19	1	20	0	12	9
	Not tested/not evaluable	8	49	41	21	30	33
IHC	IHC negative (H-score < 120)	2	28	20	9	13	21
	IHC positive (H-score > 120)	19	23	26	14	19	14
	Not tested	6	16	30	4	20	14

GIM, Genomic Instability Metric; TMB, tumor mutational burden; LOH, loss of heterozygosity; MSI, microsatellite instability; FISH, fluorescence *in situ* hybridization; IHC, immunohistochemistry. *pathogenic mutations.

### GIM effective proxy for outcome prediction

Follow-up data were available in 68 patients (66.67%). All but one of these patients were treated with first-line platinum-based CT, which was referred to as best supportive care for poor performance status and thus excluded from further analyses. Two patients were indeed excluded from the survival analysis for having stage I and II disease. After completing CT, maintenance therapy was administered in 47 patients (69.11%), with bevacizumab (9/68 cases, 13.23%), PARPi (27/68, 39.70%), or both (11/68, 16.18%). In 10 patients, maintenance therapy was not administered due to disease progression or death while receiving CT, none of them carrying a *BRCA1/2* pathogenic mutation or positive GIM. Among the 19 patients with pathogenic/likely pathogenic *BRCA1/2* mutations, maintenance therapy was administered in 15 (78.95%), with PARPi (13) or PARPi plus bevacizumab (2). Among the 49 *BRCA1/2* wild-type patients, maintenance therapy was administered in 32 (65.30%), with PARPi (14), PARPi plus bevacizumab (9), and bevacizumab (9). Median follow-up was 27.60 months. NGS testing was performed at disease onset in 66/68 cases (97.1%), whereas the remaining two cases (2.9%) were profiled at relapse following platinum-based CT.

**Figure 6:**
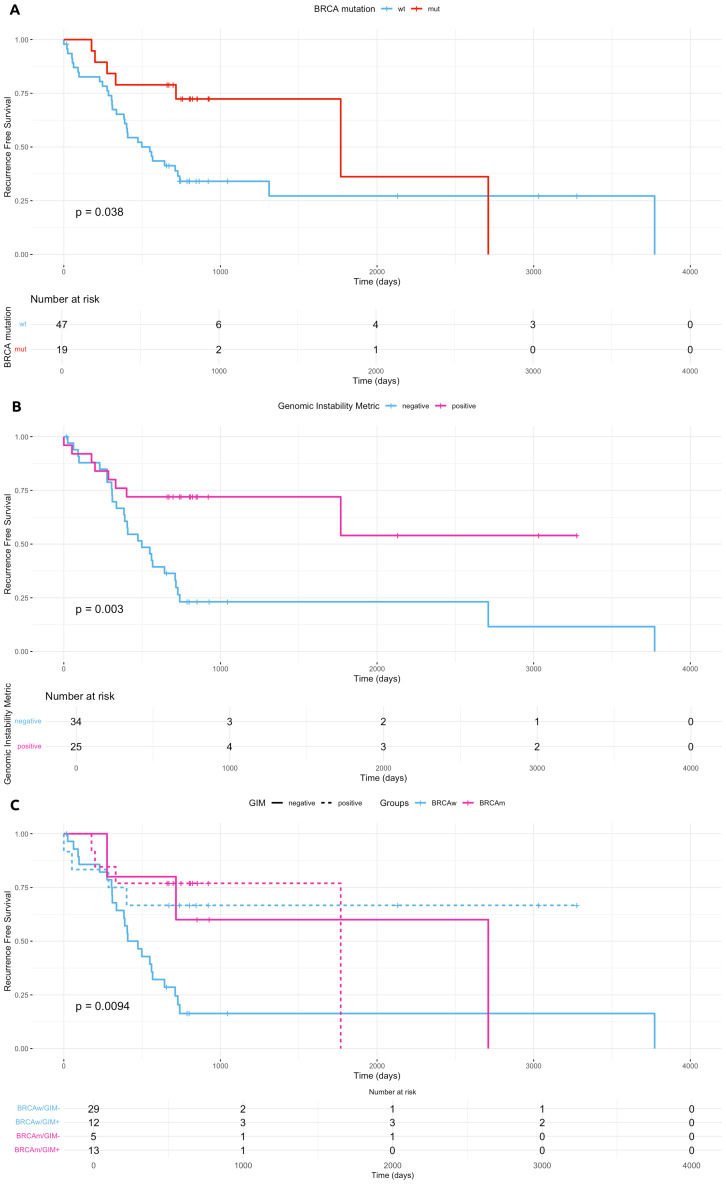
Clinical outcomes. **(A–C)** Kaplan–Meier estimates of recurrence-free survival according to *BRCA1/2* mutational status **(A)**, homologous recombination status calculated with GIM score **(B)**, and both **(C)**. Panel **(C)** confirms that patients with GIM-positive scores had a similar outcome independent of *BRCA*1/2 mutation presence. wt, wild type; mut, mutated; BRCAm, *BRCA*1/2 mutated; BRCAwt, *BRCA*1/2 wild type; GIM, Genomic Instability Metric.

HGSC patients carrying *BRCA1/2* mutation and treated with platinum-based CT showed a better RFS (p = 0.038, **Figure 6A**). Interestingly, patients with a positive GIM score had a similar outcome (p = 0.003, **Figure 6B**). Likewise, *BRCA1/2* mutations (OR = 0.30, CI = 0.098–0.913) and GIM score (OR = 0.13, CI = 0.040–0.408) were significantly associated with a lower risk of disease recurrence. Patients with GIM-positive high-grade serous ovarian carcinoma (HGSOC) had a similar outcome independent of *BRCA1/2* mutation status, with median RFS of 18.73 and 58.12 months for positive and negative GIM, respectively. GIM negative/*BRCA1–2* wild-type patients had a worse PFS compared to both *BRCA1/2*-mutated and GIM-positive patients (p = 0.0094, OR for recurrence = 0.19, CI = 0.062–0.557, **Figure 6C**). Moreover, in the *BRCA1/2* wild-type cohort, no difference in RFS was observed according to maintenance regimen (PARPi, bevacizumab, or both; **Supplementary Figure S5**).

*CCNE1* status was not associated with platinum-based CT RFS (p = 0.9 for amplification by NGS and FISH, **Supplementary Figure S4**; p = 0.17 for overexpression by IHC, **Supplementary Figure S4**). Similarly, no association was found between RFS and *CCNE1* amplification or overexpression.

## Discussion

We report an extensive genomic analysis of a real-world cohort of HGSC patients carried out with the Thermo Fisher Oncomine Comprehensive Plus panel (OCA Plus). Among the different analyses, OCA Plus provides a calculation of the level of HRD (the GIM score), particularly important since it has recently become a target for PARPi therapy. Previous studies have performed a technical validation of the GIM score (15): Shejbel et al. found a concordance of 89% (60% in samples with an estimated tumor fraction < 30%) between OCA Plus and Myriad tests in a cohort of 80 HGSC patients. In our cohort, the OCA Plus GIM score was positive in 40% of cases, with half of them (20% of the overall cohort) carrying *BRCA1/2* pathogenic mutations as expected (p = 0.009). We found that GIM-positive patients bore alterations in several HRR genes, with *BRCA1/2*, *BRIP1*, *ATM*, *POLE*, *POLD1*, *CDK12*, *PPP2R2*, *NBN*, *RAD54L*, *RAD51C*, and *RAD51B* being the most frequent. Notably, GIM-positive cases also had a high LOH, further sustaining the link between genomic instability and high LOH levels. LOH percentage measurement is closely aligned with the LOH score calculated using FoundationOne Assay, which was used for assessing HRD status and PARPi treatment in the ARIEL2 phase 2 clinical trial (36).

In our cohort, eight patients (29.63% of *BRCA1/2*-mutated) carrying pathogenic variants in *BRCA1/2* had a negative GIM value. Although these cases represent a minority, they are particularly noteworthy, as HRD is primarily associated with *BRCA1/2* mutations (6), making their negative GIM scores an unexpected and biologically relevant observation. While HRD can frequently occur in the absence of *BRCA1/2* mutations, the opposite—*BRCA1/2* mutations without detectable HRD—is considered much rarer, further highlighting the peculiarity of these cases. As proposed by Shejbel et al. (15), this finding could have potential technical explanations, mainly related to tumor fraction estimates or values very close to the positivity threshold. However, alternative biological mechanisms may also be involved, including epigenetic mechanisms (reversion of methylated promoters or overexpression of miRNA) or the activation of alternative pathways (37). As mentioned, data on the technical validation of the GIM score have already been extensively published in the literature, but its clinical validity is less well documented. Consistently, our real-world clinical data showed comparable RFS trends in HRD patients to those obtained with the reference test Myriad, further supporting its utility in informing therapeutic decisions for HGSC patients. Similarly, with the limitation due to the short median follow-up of our series (28 months) compared to the PAOLA1 trial (60 months), we showed that *BRCA1/2* wt-HR-proficient patients, as determined by OCA Plus or Myriad assays, had similar RFS. The interchangeability of different tests for HRD suggests that there is an urgent need to harmonize these assays in pathology laboratories.

We found *CCNE1* amplification in 29% of our cases, negatively correlated with *BRCA1/2* mutations. When present, *CCNE1* amplification is an early genomic event in HGSC that is maintained throughout the clinical course of the disease (38), and the high prevalence of this alteration in our cohort, particularly in tumors lacking *BRCA1/2* mutations, underscores its potential role as an independent oncogenic driver. HGSCs harboring *CCNE1* amplification are typically associated with chemoresistance and poor prognosis (39); however, this alteration is increasingly recognized as a promising predictive biomarker for emerging molecularly targeted therapies (22–24).

While we found a high concordance between NGS- and FISH-detected amplification, in testing CCNE1 expression by IHC, we observed, surprisingly, high levels of immunoreactivity in nearly half of the cases in the absence of gene amplification. These data were confirmed by a significant but mild correlation between NGS copy numbers and IHC H-score values, which could be caused, in our opinion, by the large number of non-amplified CCNE1-expressing cases. This behavior has been previously reported in literature (40, 41) and has been proposed to be caused by mutations or methylation in ubiquitin–proteasome pathway genes, which impair protein degradation, eventually resulting in CCNE1 accumulation. Our data confirmed that no molecular differences seemed to exist between non-amplified CCNE1-expressing and non-expressing patients. We considered the possibility that the protein enrichment observed in non-amplified samples could be related to low-level *CCNE1* gains. However, this hypothesis was not confirmed by our data, leading us to believe that an alternative mechanism at the transcriptional or epigenetic level may actually account for this phenomenon. These findings are, in our opinion, one of the most promising results of this study because they underline the importance of implementing IHC, possibly integrated with RNA sequencing, in the clinical assessment of *CCNE1* alterations for molecularly driven therapies. Notably, the phase I clinical trial NCT05238922, recruiting patients who are positive for CCNE1 by IHC, is currently active in several countries (42). Preliminary results from trials enrolling patients based on either amplification or immunohistochemistry data suggest that response appears to correlate more strongly with protein expression than with gene amplification. These findings indicate that relying solely on *CCNE1* amplification may underestimate the population of patients potentially sensitive to the drug (43). In our view, this could be partly explained by the particular behavior of the protein discussed above. However, as *CCNE1* is a relatively new biomarker, the issue remains unresolved. Currently, neither the antibody nor the IHC cut-off values are standardized and are still under evaluation, and the degree to which amplification serves as a reliable surrogate for protein expression is still unclear.

Targetable mutations rarely appeared with *BRCA1/2* alterations, HRD, or *CCNE1* amplification. Therefore, it was not possible to formulate hypotheses or perform meaningful statistical analyses regarding co-occurring alterations. On the contrary, we observed that approximately 8/50 of *CCNE1*-negative HR-proficient patients (7.84 overall) carried pathogenic alterations in the *PTEN*, *PIK3CA*, *BRAF*, *KRAS*, or *CDK12* genes, being potentially eligible for off-label treatments.

Our data suggest that using CGP in OC patients is effective in capturing *BRCA1/2* status and complex metrics like HRD, as well as in identifying biomarkers exploitable in clinical trials for *BRCA* wild-type and HR-proficient patients. We therefore believe that performing a comprehensive NGS test that includes all actionable biomarkers at an early stage could indeed represent an advantageous up-front strategy, offering improved clinical management, time saving, and tissue preservation for patients, as well as cost-effectiveness for healthcare systems. Neverthless, the primary aim remains to achieve a more refined and accurate stratification of HGSC patients, enabling better clinical decision-making, and in this context, molecular tumor boards (MTBs) represent the ideal tool for governing modern precision oncology (29, 44). Indeed, the compliance with MTB recommendations has been associated with a better outcome for women with gynecological cancers and breast cancer (45, 46).

In conclusion, we demonstrated the utility of CGP for patients with HGSC in a real-world setting, highlighting its analytical validity and the potential to reliably identify new biomarkers for novel molecularly driven treatments.

## Data Availability

The raw data supporting the conclusions of this article will be made available by the authors, without undue reservation.

## Supplementary material

Supplementary Figure 1 barplot showing the distribution of CCNE1 staining H-score used for the discriminating threshold assessment.

Supplementary Figure 2 Boxplot showing the positive correlation between a positive value of genomic instability metric (GIM) and LOH percentage.

Supplementary Figure 3 Visualizing of the correlations between the principal molecular markers. Boxplots showing the comparisons of *Ccne1* amplification status with molecular markers detected by NGS in OCA plus panel. Respectively: TMB (tumor mutational burden), total mutations number detected per sample, GIM and LOH%.

Supplementary Figure 4 Clinical outcomes. **(A, B)** Kaplan-Meier estimates of recurrence free survivals according to *Ccne1* amplification **(A)** and expression **(B)** status.

Supplementary Figure 5 Clinical outcomes. Kaplan-Meier estimates of recurrence free survivals in the three different maintenance treatment groups. Bev: Bevacizumab; Bev-PARPi: Bevacizumab plus PARP-inhibitors; PARPi: PARP-inhibitors.

Supplementary Table 1 The copy numbers values estimed respectively for NGS and FISH. NGS values were calculated by Ion Reporter Software, FISH values have been approximated according to estimates calculated as described in Result section and in ref 35.
