# Supplementary figures and images for: Genomic instability and CCNE1 amplification as emerging biomarkers for stratifying high-grade serous ovarian cancer
Source: Front Oncol. 2025 Aug 6;15:1633410. doi: 10.3389/fonc.2025.1633410 (PMC12364670; doi:10.3389/fonc.2025.1633410)

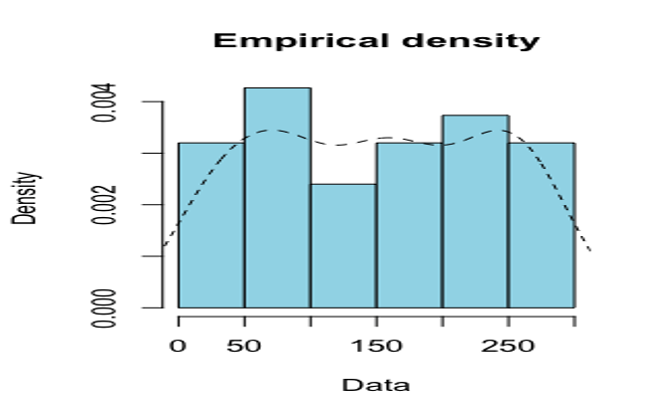

Supplement: Supplementary Figure 1 — barplot showing the distribution of CCNE1 staining H-score used for the discriminating threshold assessment. [file Image1.tif]

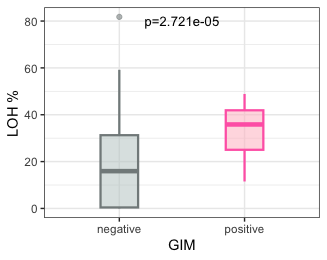

Supplement: Supplementary Figure 2 — Boxplot showing the positive correlation between a positive value of genomic instability metric (GIM) and LOH percentage. [file Image2.tif]

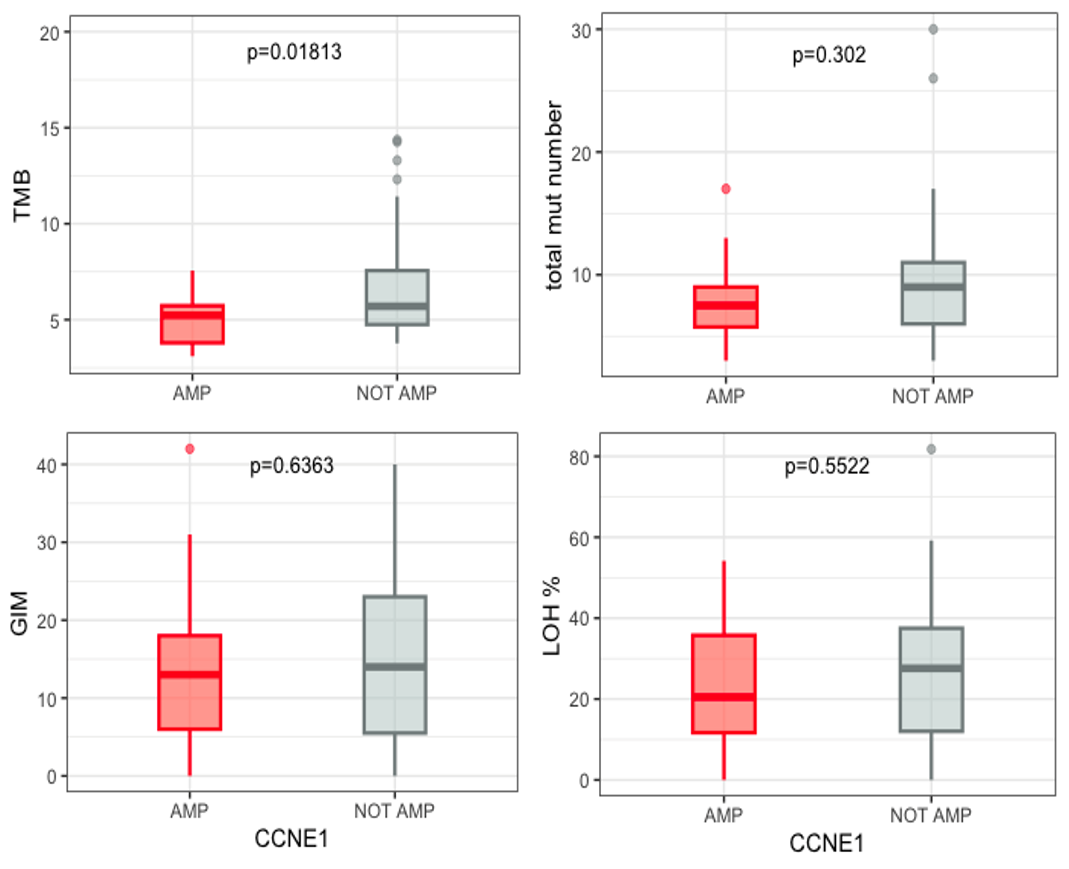

Supplement: Supplementary Figure 3 — Visualizing of the correlations between the principal molecular markers. Boxplots showing the comparisons of Ccne1 amplification status with molecular markers detected by NGS in OCA plus panel. Respectively: TMB (tumor mutational burden), total mutations number detected per sample, GIM and LOH%. [file Image3.tif]

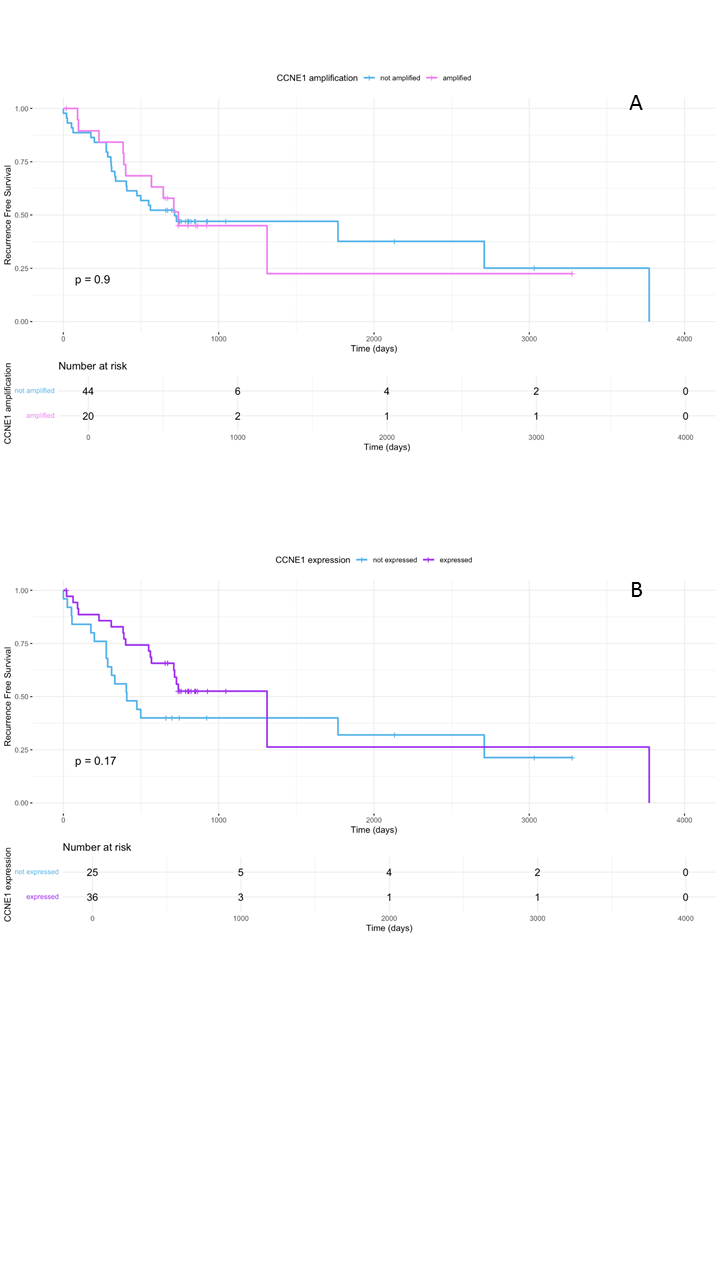

Supplement: Supplementary Figure 4 — Clinical outcomes. (A, B) Kaplan-Meier estimates of recurrence free survivals according to Ccne1 amplification (A) and expression (B) status. [file Image4.tif]

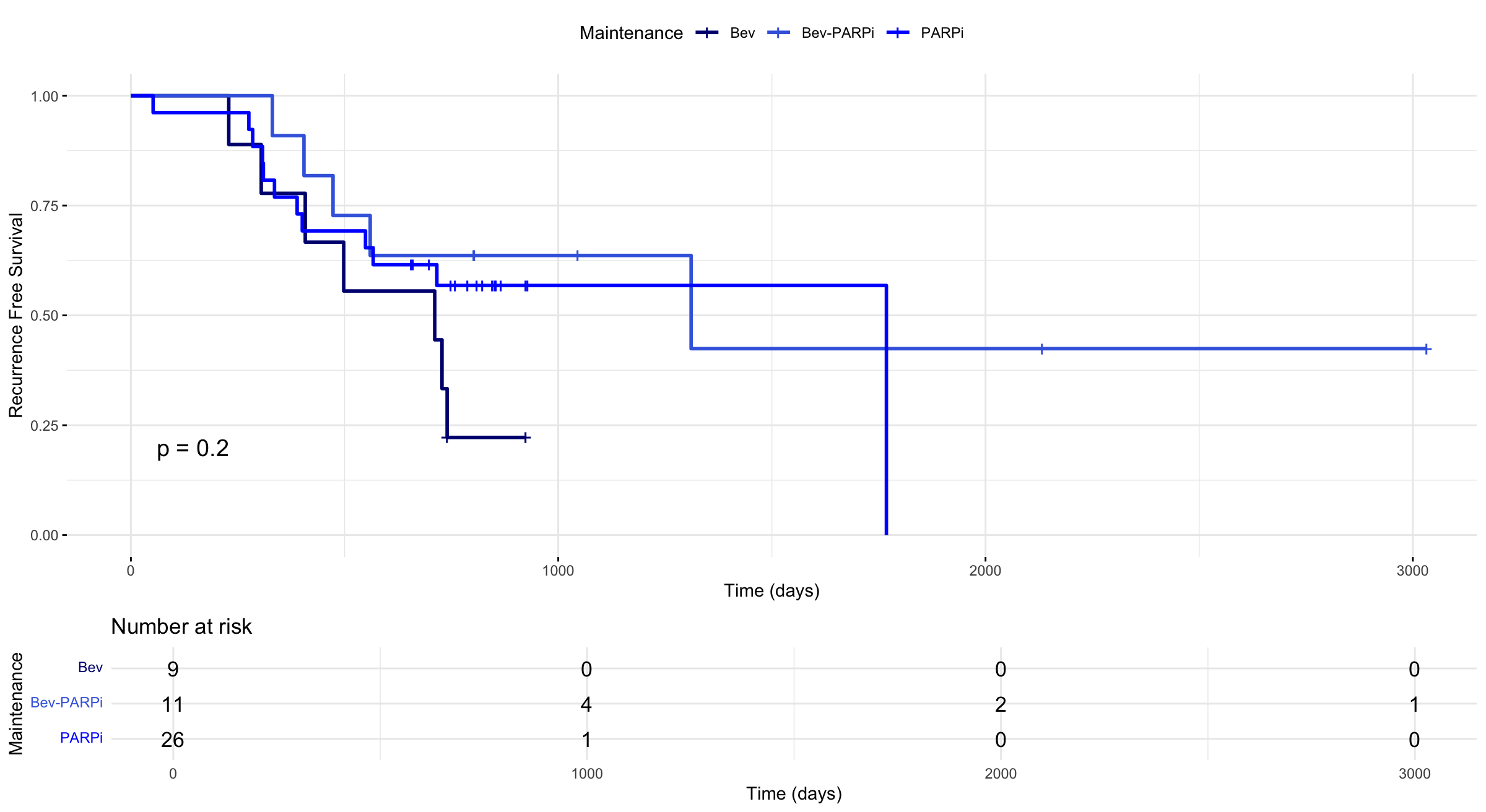

Supplement: Supplementary Figure 5 — Clinical outcomes. Kaplan-Meier estimates of recurrence free survivals in the three different maintenance treatment groups. Bev: Bevacizumab; Bev-PARPi: Bevacizumab plus PARP-inhibitors; PARPi: PARP-inhibitors. [file Image5.tif]
